# Supplementary material for: Cohort profile: The UK COVID-19 Public Experiences (COPE) prospective longitudinal mixed-methods study of health and well-being during the SARSCoV2 coronavirus pandemic
Source: PLoS One. 2021 Oct 13;16(10):e0258484. doi: 10.1371/journal.pone.0258484 (PMC8513913; doi:10.1371/journal.pone.0258484)
Supplement: S1 File — (PDF) [file pone.0258484.s001.pdf]

UK public experiences during the coronavirus COVID19 pandemic

Page 1: Page 1

We are carrying out a survey to find out about the experiences of people living in the UK during the coronavirus COVID19 pandemic. We are interested in what members of the public think, feel, and do during the pandemic. The survey is open to anyone aged 18 years and over who is currently living in the UK. Please click here for full information on the study:

https://static.onlinesurveys.ac.uk/media/account/3759/survey/578689/question/covid_19_information_sheet_onl.docx

Page 2: Consent to take part in the survey

Please confirm the following information:

- I am currently living in the UK
- I am 18 years of age or over
- I have read the information sheet provided
- I understand that my participation is completely voluntary
- I would like to take part in this survey

1. Do you confirm that the above information is correct, and that you would like to take part in this survey?

- ◯ Yes
- ◯ No

If you answered **no** to any of these statements, please do not continue with this survey. For current up-to-date information on the coronavirus COVID-19 infection in the UK, please visit: https://111.nhs.uk/covid-19 · https://www.nhs.uk/conditions/coronavirus-covid-19/ · https://www.gov.uk/guidance/coronavirus-covid-19-information-for-the-public

Page 3: About you

Thank you for taking part in this survey. Firstly, we would like to ask you a few questions about yourself and your general health.

2. What is your age group?

- ○ 18 to 30 years
- ○ 31 to 40 years
- ○ 41 to 50 years
- ○ 51 to 60 years
- ○ 61 to 70 years
- ○ 71 to 80 years
- ○ 81+ years
- ○ Rather not say

3. Which gender do you identify with?

- ○ Male
- ○ Female
- ○ Prefer to self-describe
- ○ Rather not say

4. Is this the same gender you were assigned at birth?

- ○ Yes
- ○ No
- ○ Rather not say

5. Do you have any pre-existing medical conditions? Please tick all that apply.

- ☐ None
- ☐ Cardio-vascular condition

- [ ] Respiratory condition
- [ ] Diabetes
- [ ] Cancer
- [ ] Other

5.a. If you selected Other, please specify:

6. Have you been vaccinated against seasonal flu during the last 12 months?

- ○ Yes
- ○ No
- ○ Don't know

7. Do you have children (younger than 18 years) in your household?

- ○ Yes
- ○ No
- ○ Rather not say

7.a. Are any of your children under 5 years of age?

- ○ Yes
- ○ No
- ○ Rather not say

7.b. Do any of your children have pre-existing health conditions?

- ○ Yes
- ○ No
- ○ Rather not say

7.b.i. Please tell us what these pre-existing conditions are.

8. Do you have caring responsibilities for any adults who are over 70 years of age?

- Yes
- No
- Rather not say

9. Do you have caring responsibilities for any adults who have pre-existing health conditions?

- Yes
- No
- Rather not say

Page 4: Your experiences of the COVID19 pandemic

This section of the survey is about the current coronavirus COVID-19 pandemic. Please base your answers on what you currently know now about the pandemic.

Please remember that all the answers you provide will be kept completely confidential. You can skip any questions that you would rather not answer.

10. Do you think that the COVID-19 pandemic has had any impact on your day-to-day life so far? If so, please tell us what impact it has had.

11. Have you done anything so far in response to COVID-19 to try to avoid getting the infection? If so, what have you done?

12. Have you done anything so far to prepare for COVID-19 prevention measures that might come in, such as closing schools or limiting travel? If so, please tell us how you have prepared.

13. Do you think you have already had, or currently have COVID-19?

- Yes
- Maybe
- No
- Rather not say

13.a. Have you had some of the symptoms of COVID-19 (e.g. fever, cough, difficulty breathing)?

- Yes
- No
- Rather not say

13.a.i. Have you been diagnosed with COVID-19 by a health professional?

- Yes
- No
- Rather not say

13.a.i.a. Have you had a laboratory test to see if you have COVID-19?

- Yes - the test was positive
- Yes - the test was negative
- No
- Don't know
- Rather not say

14. Have any of your family, close friends, colleagues, or other people with whom you have regular physical contact with been diagnosed with COVID-19?

- Yes
- No
- Don't know
- Rather not say

15. Has anyone in your local area (within about 30 miles of your home or place of work/study) been diagnosed with COVID-19?

- Yes
- No
- Don't know

- Rather not say

16. Based on what we know now, do you think the following statements are true or false?

	True	False	Don't know
COVID-19 is caused by a new virus that is part of the ‘coronavirus’ group of viruses	○	○	○
A vaccine is not yet available for the COVID-19 virus	○	○	○
COVID-19 can be transmitted by human-to-human contact	○	○	○
People have died from COVID-19	○	○	○
A lot of people already have immunity to COVID-19	○	○	○
Symptoms of COVID-19 are always visible	○	○	○
You can only catch COVID-19 once	○	○	○
A major flu or cold pandemic happens about once in every 10-50 years	○	○	○
Usual common colds can be caused by certain types of coronavirus that we already know about	○	○	○

17. How severe do you think the Coronavirus COVID-19 infection will be in the UK general population as a whole?

- Very mild
- Mild
- Moderately severe
- Very severe
- Extremely severe

18. How likely do you think it is that you will get these conditions in the next 12 months?

	Very unlikely	Fairly unlikely	Fairly likely	Very likely
Seasonal influenza (flu)	○	○	○	○
Swine flu (H1N1)	○	○	○	○
Common cold	○	○	○	○
Coronavirus COVID-19	○	○	○	○

Measles	○	○	○	○
A stomach bug (i.e. sickness and diarrhoea)	○	○	○	○

19. Please indicate how harmful you think it would be to your health if you were to get the following conditions in the next 12 months.

	Not harmful at all	Slightly harmful	Somewhat harmful	Very harmful
Seasonal influenza (flu)	○	○	○	○
Swine flu (H1N1)	○	○	○	○
Common cold	○	○	○	○
Coronavirus COVID-19	○	○	○	○
Measles	○	○	○	○
A stomach bug (i.e. sickness and diarrhoea)	○	○	○	○

20.

	Not at all	A bit	Fairly	Very
Are you scared of Coronavirus COVID-19?	○	○	○	○
Are you worried about Coronavirus COVID-19?	○	○	○	○

21. How often do you think about coronavirus COVID19?

- ○ Never
- ○ Occasionally
- ○ Often
- ○ Very often
- ○ All the time

22. If you are worried about coronavirus COVID19, please tell us what your main concerns are.

Page 5: Coronavirus COVID19

23. These are some things that people might do to reduce their risks of infection. Please tell us whether you are doing these things less often, about the same, or more often than usual at the moment.

	Less often	About the same	More often	Not applicable
Avoiding crowded places	○	○	○	○
Washing hands thoroughly	○	○	○	○
Avoiding touching your face with unwashed hands	○	○	○	○
Using hand sanitiser/alcohol gel	○	○	○	○
Wearing face masks/surgical masks	○	○	○	○
Avoiding regions with coronavirus COVID-19	○	○	○	○
Avoiding people who have coronavirus COVID-19	○	○	○	○
Avoiding shaking hands and close physical contact with others, even if they appear healthy	○	○	○	○
Staying home from school or work	○	○	○	○
Seeking advice from NHS 111	○	○	○	○

24. Please indicate how **effective** you think each of these measures would be in **preventing you** from getting Coronavirus COVID-19.

	Not at all effective	A little effective	Fairly effective	Very effective
Avoiding crowded places	○	○	○	○
Washing hands thoroughly	○	○	○	○
Avoiding touching your face with unwashed hands	○	○	○	○
Using hand sanitiser/alcohol gel	○	○	○	○
Wearing face masks/surgical masks	○	○	○	○
Avoiding regions/persons with Coronavirus COVID-19	○	○	○	○
Stopping people travelling to and from Coronavirus COVID-19 affected areas	○	○	○	○
Avoiding shaking hands and close physical contact with others, even if they appear healthy	○	○	○	○
Staying home from school or work	○	○	○	○
Seeking advice from NHS 111	○	○	○	○

25. Is there anything else that you think could do to avoid getting Coronavirus COVID-19?

26. For each measure, please indicate if you think **you would be able to do this** if the government or health authorities advise you to.

	How able would you be to do this?		
	Definitely not	Maybe	Definitely would
Avoiding crowded places	○	○	○
Washing hands thoroughly	○	○	○
Avoiding touching your face with unwashed hands	○	○	○
Using hand sanitiser/alcohol gel	○	○	○
Wearing face masks/surgical masks	○	○	○
Avoiding regions/persons with Coronavirus COVID-19	○	○	○
Avoiding shaking hands and close physical contact with others, even if they appear healthy	○	○	○
Staying home from school or work	○	○	○
Seeking advice from NHS 111 if you get symptoms of COVID-19	○	○	○

26.a. If you are uable to do any of the things listed in this question please tell us more about why this would be difficult for you.

27. Is there anything that would help you follow advice from government or health authorities on COVID-19 (e.g. financial help, protection of jobs, practical things to help you manage pre-existing conditions, emotional or social support)?

Page 6: Information about COVID19

28. Do you think you have had enough information about reducing your risk of catching COVID-19?

- Yes
- No

29. Do you think you have had enough information about preparing for government responses that might come in to help manage the COVID-19 pandemic (e.g. school closure, travel restrictions)?

- Yes
- No

30. If you don’t think you have had enough information about COVID-19, what additional information would you like?

31. Where have you received information about Coronavirus COVID-19 from? Please tick all that apply.

- The NHS 111 service
- From a health professional
- Friends and family
- Social media (e.g. Twitter, Facebook, YouTube, Instagram)
- Newspapers
- Television
- Radio
- Through your place of work or study
- Official health agency websites (e.g. NHS, Public Health England, Public Health Wales, World Health Organisation, CDC)
- Other

31.a. If you selected Other, please specify:

32. Do you think that information about Coronavirus COVID-19 from the following sources is reliable?

	Not reliable at all	A little reliable	Fairly reliable	Very reliable
Government				
Local and national health agencies (e.g. the NHS)				
International health agencies (e.g. the World Health Organisation)				
Social media				
Television				
Newspapers				
Radio				

33. Please indicate the degree to which you agree with each of these statements about the COVID-19 pandemic.

	Strongly disagree	Disagree	Neither agree nor disagree	Agree	Strongly agree
There is nothing we can do about it					
The threat is exaggerated by media and government					
The government is not responding quickly enough					
It will not be as bad as predicted					
The NHS is well prepared to deal with COVID-19					
The government is doing enough to prevent the spread of COVID-19					
We will all be completely powerless					
We just have to accept it					

34. Has the coronavirus had an effect on any of the following activities? Please tell us whether you have done these things more, about the same, or less than you usually would over the last month.

	More than usual	About the same as usual	Less than usual	Not applicable
Work	○	○	○	○
Study	○	○	○	○
Travelling within the UK	○	○	○	○
Travelling outside of the UK	○	○	○	○
Getting enough rest	○	○	○	○
Spending time outdoors	○	○	○	○
Being physically active/exercising	○	○	○	○
Socialising	○	○	○	○
Healthy eating	○	○	○	○
Taking vitamin supplements	○	○	○	○
Keeping warm	○	○	○	○
Smoking	○	○	○	○
Drinking alcohol	○	○	○	○
Doing activities that you find relaxing	○	○	○	○

35. Is there anything else that you would like to tell us about your experience of the COVID-19 pandemic so far?

Page 7: About you and your health

In this section, we would like to ask you more about yourself and your general health and well-being so that we can understand more about who has completed our survey. Please remember that you can leave any questions that you don't want to answer.

36. In general, would you say your health is:

- Excellent
- Very good
- Good
- Fair
- Poor

37. These questions are about how you have been feeling during the past 4 weeks. For each question, please give one answer that comes closest to the way you have been feeling. How much of the time during **the past 4 weeks:**

	All of the time	Most of the time	A good bit of the time	Some of the time	A little of the time	None of the time
Have you felt calm and peaceful?						
Did you have a lot of energy?						
Have you felt downhearted and blue?						

Page 8: About you and your health

38. Have you travelled outside of the UK in the last month?

- Yes
- No
- Rather not say

38.a. Which county or countries have you travelled to within the last month?

39. Do you work in any of the following sectors? Please tick all that apply.

- Schools or universities
- Healthcare settings (e.g. clinics, hospitals, screening services, public health)
- Government agencies
- Frontline emergency (999) services
- Energy or telecommunications companies
- Retail of food and/or household goods
- None of the above

40. Are you able to do some or all of your work from home if you need to?

- Yes - most or all of my work can be done from home
- Yes - some of my work can be done from home
- No

41. If health agencies or the government advised you not to go to work, or your workplace was closed temporarily, would you be entitled to any pay? Please tick one answer.

- Full pay

- Partial pay
- Employer's sick pay
- Statutory sick pay
- No pay
- Don't know

42. What is your current marital status?

- Single
- Married or in civil partnership
- Living with partner
- Widowed, divorced, or separated
- Other
- Rather not say

43. What is your highest educational qualification? (Please tick one option)

- Usual high school qualifications in your country at age 16 (e.g. GCSE, O-level)
- Usual high school qualifications in your country at age 18 (E.g. AS level, A-Level)
- A college or university diploma or degree
- A higher degree or professional qualification (e.g. a Doctorate or Masters level degree)
- None of these qualifications
- Other
- Rather not say

44. How would you describe your current employment status? (Please tick all that apply)

- In full-time paid work, as an employee or self-employed
- In part-time paid work, as an employee or self-employed
- Unemployed and seeking work
- Not employed and not currently seeking work
- In full-time education or training
- In part-time education or training

- [ ] Retired
- [ ] Rather not say

45. Is the UK your country of birth?

- ( ) Yes
- ( ) No
- ( ) Rather not say

45.a. What is your country of birth?

____

46. Please choose one option that best describes your ethnic group or background:

- ( ) White (English/Welsh/Scottish/Northern Irish/British)
- ( ) White - other
- ( ) Black/African/Caribbean/Black British
- ( ) Asian/Asian British
- ( ) Mixed/Multiple ethnic groups
- ( ) Other
- ( ) Rather not say

47. Do you have a religion?

- ( ) No religion
- ( ) Christian (all denominations)
- ( ) Buddhist
- ( ) Hindu
- ( ) Jewish
- ( ) Muslim
- ( ) Sikh
- ( ) Rather not say

- Other

47.a. If you selected Other, please specify:

[ ]

48. Which of these options best describe how you think of yourself?

- Heterosexual/straight
- Gay/Lesbian
- Bisexual
- Other
- Rather not say

Page 9: What happens next

Thank you for taking part in this survey.

IF YOU THINK YOU HAVE SYMPTOMS OF CORONAVIRUS PLEASE CONTACT NHS BY PHONE ON 111 FOR ADVICE.

49. We would like to find out more about people's experiences over the next 12 months as the coronavirus COVID-19 pandemic develops. Would you be willing for us to contact you about:

	Yes	No
Follow-up online surveys in three and twelve months time	○	○
Taking part in an informal interview about your experiences over the phone	○	○

If you have answered yes to either of these questions, please provide us with your e-mail address and/or mobile phone number. *Your contact details will be kept completely confidential. These will be used by our research team only for the specific purposes stated above. We will not share this information with any third parties.*

49.a. Mobile phone number:

49.b. E-mail address:

Page 10: Thank you

Thank you for taking part.

For current up-to-date information on the coronavirus COVID-19 infection in the UK, please visit:

- https://111.nhs.uk/covid-19
- https://www.nhs.uk/conditions/coronavirus-covid-19/
- https://www.gov.uk/guidance/coronavirus-covid-19-information-for-the-public

If you would like support with any of the issues raised in the questionnaire, you can contact:

- Your GP or another member of your healthcare team
- Mind – promotes the views and needs of people with mental health problems. Phone: 0300 123 3393 (Monday to Friday, 9am to 6pm), website: www.mind.org.uk

Further information

If you have any questions about the research or how we intend to conduct the study, please contact:

Dr Rhiannon Phillips

Cardiff School of Sport & Health Science

Cardiff Metropolitan University

Llandaff Campus

Cardiff CF5 2YB

Phone: 02920 416893

E-mail: COVID19publicsurvey@cardiffmet.ac.uk
