# Supplementary material for: Cohort profile: The UK COVID-19 Public Experiences (COPE) prospective longitudinal mixed-methods study of health and well-being during the SARSCoV2 coronavirus pandemic
Source: PLoS One. 2021 Oct 13;16(10):e0258484. doi: 10.1371/journal.pone.0258484 (PMC8513913; doi:10.1371/journal.pone.0258484)
Supplement: S1 Table — (DOCX) [file pone.0258484.s003.docx]

**Table S3:** **Baseline survey demographics for all participants, and participants recruited via the HWW (Wales only) and SM (UK-wide) recruitment routes**

| **Characteristic** | **Category** | **All** | | **HWW recruitment route** | | **SM recruitment route** | |
| --- | --- | --- | --- | --- | --- | --- | --- |
|  |  | **n** | **%** | **n** | **%** | **n** | **%** |
| COPE baseline participants |  | 11,113 | 100 | 8,726 | 79 | 2,386 | 21 |
| Gender | Male | 3,359 | 30.2 | 2,950 | 33.8 | 409 | 17.1 |
|  | Female | 7,694 | 69.2 | 5,739 | 65.8 | 1,955 | 81.9 |
|  | Prefer to self-describe | 20 | 0.2 | 12 | 0.1 | 8 | 0.3 |
|  | Missing/rather not say | 40 | 0.4 | 26 | 0.2 | 14 | 0.6 |
| Age group | 18 to 30 years | 810 | 7 | 434 | 5 | 376 | 15.8 |
|  | 31 to 40 years | 1,251 | 11 | 794 | 9.1 | 457 | 19.2 |
|  | 41 to 50 years | 1,459 | 13 | 936 | 10.7 | 523 | 21.9 |
|  | 51 to 60 years | 2,352 | 21 | 1,798 | 20.6 | 554 | 23.2 |
|  | 61 to 70 years | 3,229 | 29 | 2,848 | 32.6 | 381 | 16 |
|  | 71 to 80 years | 1,786 | 16 | 1,701 | 19.5 | 85 | 3.6 |
|  | 81+ years | 211 | 2 | 208 | 2.4 | <5 | <1 |
|  | Rather not say / Missing | 15 | 0.1 | 8 | 0 | 7 | 0 |
| Ethnicity | White | 10,830 | 97.5 | 8,553 | 98 | 2,277 | 95.4 |
|  | Minority ethnic communities | 165 | 1.5 | 87 | 1 | 78 | 3.3 |
|  | Missing | 118 | 1 | 87 | 1 | 31 | 1.4 |
| Relationship status | Single | 1,638 | 14.7 | 1,178 | 13.5 | 460 | 19 |
|  | Married, in a civil partnership, or living with partner | 7,569 | 68.1 | 5,940 | 68.1 | 1,629 | 68 |
|  | Widowed, divorced or separated | 1,722 | 15.5 | 1,492 | 17.1 | 230 | 9.6 |
|  | Other | 92 | 0.8 | 56 | 0.6 | 36 | 1.5 |
|  | Missing or rather not say | 92 | 0.8 | 61 | 0.7 | 31 | 1.3 |
| Caring responsibilities | No caring responsibilities | 6,469 | 58.2 | 5,347 | 61.3 | 1,122 | 47 |
|  | At least one caring responsibility | 4,644 | 41.8 | 3,380 | 38.7 | 1,264 | 53 |
|  | Had children under age 18 in household | 2,161 | 19 | 1,355 | 16 | 806 | 34 |
|  | Had children under age 5 in household | 694 | 6 | 424 | 5 | 270 | 11 |
|  | Had children with pre-existing health condition | 440 | 4 | 285 | 3 | 155 | 7 |
|  | Had caring responsibilities for adults over age 70 | 2,096 | 19 | 1,644 | 19 | 452 | 19 |
|  | Had caring responsibilities for adults with pre-existing health condition | 2,397 | 22 | 1,889 | 22 | 508 | 21 |
| Highest level of education | Higher degree/professional qualification | 2,766 | 25 | 1,976 | 23 | 790 | 33 |
|  | A college or University diploma/degree | 4,692 | 42 | 3,796 | 44 | 896 | 38 |
|  | High school qualification age 18 (i.e. AS, A-level) | 1,066 | 10 | 805 | 9 | 261 | 11 |
|  | High school qualification age 16 (i.e. GCSE, O-level) | 1,578 | 14 | 1,300 | 15 | 278 | 12 |
|  | Other | 301 | 3 | 270 | 3 | 31 | 1 |
|  | None of these qualifications | 473 | 4 | 406 | 5 | 67 | 3 |
|  | Rather not say / Missing | 237 | 2 | 174 | 2 | 63 | 3 |
